# Variation in cystectomy pathology reporting practice—results from an international survey of 212 pathologists

**DOI:** 10.1007/s00428-024-03924-3

**Published:** 2024-09-07

**Authors:** Jon Griffin, Arndt Hartmann, Eva Comperat

**Affiliations:** 1 School of Medicine and Population Health, University of Sheffield, Sheffield, UK; 2 Histopathology Department, Sheffield Teaching Hospitals NHS Foundation Trust, Sheffield, UK; 3 Institute of Pathology, University Hospital Erlangen, Friedrich-Alexander-Universität Erlangen-Nürnberg (FAU), Erlangen, Germany; 4 Comprehensive Cancer Center EMN, University Hospital Erlangen, Friedrich-Alexander-Universität Erlangen-Nürnberg, Erlangen, Germany; 5 Bavarian Center for Cancer Research (Bayerisches Zentrum Für Krebsforschung, BZKF), Erlangen, Germany; 6 Department of Pathology, Medical University Vienna, Währingergürtel 18-20, 1090 Vienna, Austria

**Keywords:** Cystectomy, Bladder cancer, Grossing, Dissection, Neoadjuvant chemotherapy, Pathological response score

## Abstract

The pathological assessment of cystectomy specimens is important for accurate prognostic information and to inform adjuvant therapy decisions. However, there is limited evidence regarding the best approach to fixation, dissection, block selection and microscopic assessment of cystectomies. We report the results of an international survey of 212 pathologists and their approach to cystectomy pathology. There is variation at all stages of the specimen journey including in fixation and dissection techniques, and in the approach to evaluating residual tumour. This is particularly evident in the post-neoadjuvant chemotherapy setting where there is variable use of response scoring systems and differing approaches to sampling. We also find variation in the use of digital and molecular pathology in cystectomy specimens. Finally, we have suggested areas for future research in cystectomy pathological assessment.

**Supplementary Information:**

The online version contains supplementary material available at 10.1007/s00428-024-03924-3.

## Introduction

Radical cystectomy with preceding neoadjuvant cisplatin-based chemotherapy is recommended by the European Urological Association as definitive treatment for muscle-invasive bladder cancer (MIBC) in eligible patients [1]. The pathological assessment of cystectomy specimens is important for confirming the presence of remaining tumour, accurate assessment of tumour and nodal stage, diagnosing subtype histology and examination of resection margins. These parameters have impacts on post-operative/adjuvant therapy decisions. For example, the PD-L1 inhibitor Nivolumab is licensed for use in the UK for patients with ypT2 + /ypN + MIBC who have received neoadjuvant chemotherapy (NAC) or patients with pT3 + /pN + MIBC who did not have neoadjuvant treatment [2].

Pathological assessment of cystectomy specimens poses some unique challenges not encountered in other cancer resections. Most patients will have had a trans-urethral resection of bladder tumour (TURBT) as a diagnostic and potentially therapeutic procedure early in their management pathway. As a result, there may be no macroscopic tumour present in the bladder and, in approximately 10% of cases, no tumour is found microscopically (pT0) [3]. In a proportion of patients, this may be partially attributable to neoadjuvant chemotherapy; however, patients who have received solely TURBT may also achieve pT0 at cystectomy. In the post-NAC setting, an assessment of treatment effect is also necessary. Pathological down-staging to any of < ypT2, ypTis, ypTa or ypT0 has been used in clinical trials of neoadjuvant chemotherapy and, more recently, neoadjuvant immunotherapy. Downstaging correlates with survival in the neoadjuvant setting and is thus a useful surrogate end point which can give an earlier signal of treatment effect than waiting for follow-up data to mature. An attempt has been made to standardise the assessment and reporting of response to NAC in cystectomies [4, 5], similar to semi-quantitative systems used in breast and colorectal cancer. However, this approach has not seen widespread adoption in national guidelines for bladder cancer diagnosis and management.

Despite the importance of pathological assessment of cystectomy specimens, there is a surprising lack of evidence to support current approaches to practice [6]. If there is variation in practice, this could contribute to variation in the information provided by pathologists. In turn, this could affect clinically important parameters such as pathological response to NAC or accurate staging. The pathological assessment process has many points where variation can occur, from methods of fixation and dissection to tissue block selection and the use of scoring systems for treatment response. In this study, we evaluated this variation through an international survey of pathologists who report cystectomy specimens.

## Methods

We designed an 18-question survey which was distributed electronically as a Google form by the British Association of Urological Pathologists (BAUP), the International Society of Uropathology (ISUP), the Genito-Urinary Pathology Society (GUPS) and the Working group Uropathology of the German Society of Pathology (DGP) via their mailing lists. The survey was open to receive responses from 14th August to 25th September 2023 and a reminder email was sent halfway through this period. The participants were independently practicing pathologists with an interest in uropathology (consultant/attending level). All participants gave informed consent and the study received ethical approval from the University of Sheffield (UK) Ethics Committee on 24th July 2023 (approval number: 054611).

The survey questions covered practice across the entire cystectomy specimen journey and included questions about fixation methods, dissection and sampling, microscopy and molecular and digital pathology. We also asked specific questions about the assessment of cystectomies following neoadjuvant chemotherapy (NAC). The full questionnaire, study information and consent form are available in supplementary data. Questionnaire data was collected in Excel (Microsoft Corporation, Redmond, WA, USA) and analysed in R version 4.0.3 (R Foundation, Indianapolis, IN, USA) [7]. Categorical data are presented as proportional waffle plots. Upset plot is used to represent combinations of answers. Chi-squared test was used to assess statistical significance of categorical variables.

## Results

### Questionnaire cohort demographics

**Fig. 1:**
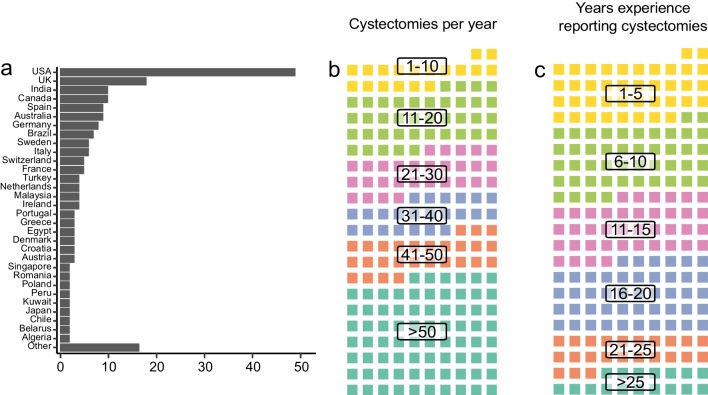
Demographics of survey respondents. (**a**) Geographical distribution of respondents. The category of ‘other’ comprises *n* = 1 each from Sri Lanka, Taiwan, Republic of Korea, Philippines, New Zealand, Norway, Myanmar, Moldova, Mexico, Luxembourg, Jordan, Guatemala, Czechia, Costa Rica, Colombia, China, Belgium and Argentina. (**b**) Proportion of respondents working in centres grouped by number of cystectomies reported annually. (**c**) Proportion of respondents grouped by years’ experience of reporting cystectomy specimens

A total of 212 pathologists from 49 countries completed the online survey. The commonest countries were USA (*n* = 49, 23%), UK (*n* = 18, 8%), India (*n* = 10, 5%) and Canada (*n* = 10, 5%) (Fig. 1a). Some 36% of pathologists were from centres that performed more than 50 cystectomies per year (Fig. 1b). Experience of reporting radical cystectomies was assessed as 5-year groupings and there was an even distribution of participants across the categories 1–5 years’ experience to 15–20 years’ experience. Altogether, these groups comprised 81% of pathologists. The remaining 19% had more than 20 years’ reporting experience (Fig. 1c). Together, these results show that the questionnaire responses captured a wide representation of geography, reporting activity and reporting experience.

### Approach to fixation and sampling of routine cystectomy specimens

**Fig. 2:**
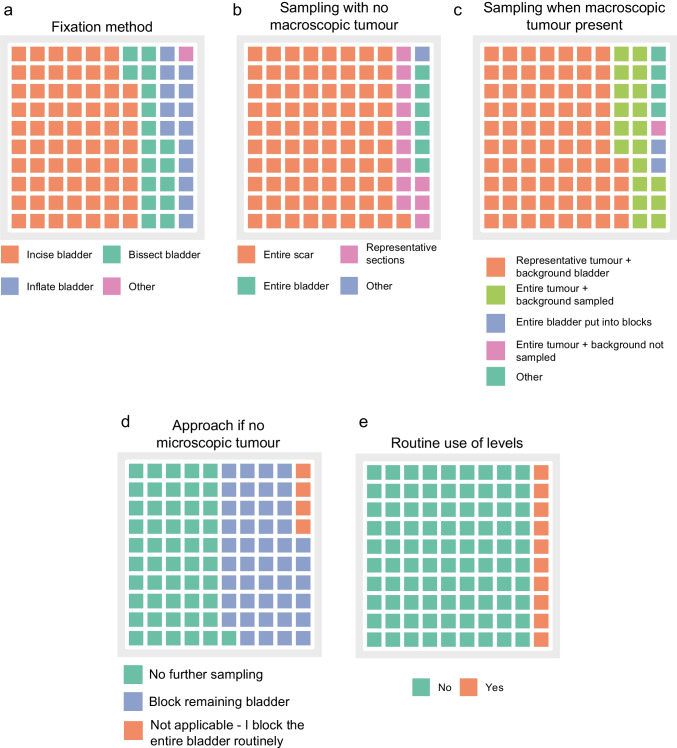
Approach to fixation and sampling of cystectomy specimens. (**a**) Method of fixation. (**b**) Pathologist approach to sampling when no macroscopic tumour was found at dissection and (**c**) when macroscopic tumour was identified. (d) Pathologist approach to taking further blocks if no tumour was identified on microscopic review of initial sampling/slides. (**e**) Pathologist use of routine levels or step sections

We next asked about pathologists’ approach to fixation, processing and sampling of cystectomies. Methods of fixation varied: 67% of respondents incised the bladder anteriorly and submerged the entire specimen in formalin to fix, 16% bisected the specimen into two halves and placed in formalin to fix and 14% inflated the bladder with formalin via the urethra (Fig. 2a). Most (93%) pathologists did not routinely perform fresh sampling of cystectomy specimens.

Two situations are commonly encountered when sampling cystectomy specimens. There may be a scarred area with no tumour present owing to previous TURBT, intra-vesical therapy and/or neoadjuvant chemotherapy. Alternatively, macroscopic tumour may remain in the bladder. We asked how pathologists approached these situations and found that, when no tumour was visible macroscopically, most respondents (81%) sampled the entire scarred area. Some 12% of respondents would take representative sections of the scar and 6% would put the entire bladder into blocks (Fig. 2b). In the second situation of a cystectomy specimen containing residual macroscopic tumour, 75% of pathologists would take representative tumour sections and sample the background bladder whereas 19% would block the entire tumour and sample the background bladder. Four respondents (2%) would block the entire bladder. The remaining nine respondents gave descriptions where their sampling strategy varied based on the size of the tumour (Fig. 2c).

Many situations in diagnostic pathology require additional work after an initial microscopic assessment of tissue sections. We recognised this as a possible scenario in the cystectomy setting when no tumour is identified in the initial tissue sampling. Respondents were asked about their approach in this context. Some 45% of pathologists would sample the rest of the bladder if their initial blocks showed no tumour. However, half of the respondents would not take this approach (Fig. 2d). To further probe microscopic assessment of cystectomies, we asked if pathologists routinely used levels or step sections to examine tissue from cystectomies in general. The majority (90%) did not routinely examine tissue at multiple levels (Fig. 2e). When asked if they used levels on a case-by-case basis, 196 pathologists gave evaluable answers. Some 64% did use levels on a case-by-case basis and listed reasons including dealing with technical issues such as requiring full-face sections, closer examination of the scarred area, assessment of margins and in cases where tumour was equivocal at a stage boundary.

### Assessment of response to neoadjuvant chemotherapy in cystectomies

Complete response to neoadjuvant chemotherapy is a good surrogate marker of cancer-specific and overall survival. Furthermore, the rate of complete response or downstaging from muscle invasive to non-muscle invasive bladder cancer is frequently used as an endpoint in neoadjuvant chemotherapy and immune-therapy trials. How response to neoadjuvant chemotherapy is assessed is therefore important as variation in assessment could impact on the predictive ability of this metric. To investigate the approach to cystectomy assessment following NAC, we first asked pathologists to estimate what proportion of patients received NAC at their institution. Interestingly, 32% of respondents did not know. Two respondents stated that patients did not receive NAC at their centre. The remaining respondents reported an approximately even distribution across the quintiles 1–20%, 21–40%, 41–60% and 61–80% of patients receiving NAC. Only 7/212 respondents indicated that greater than 80% of patients received NAC.

**Fig. 3:**
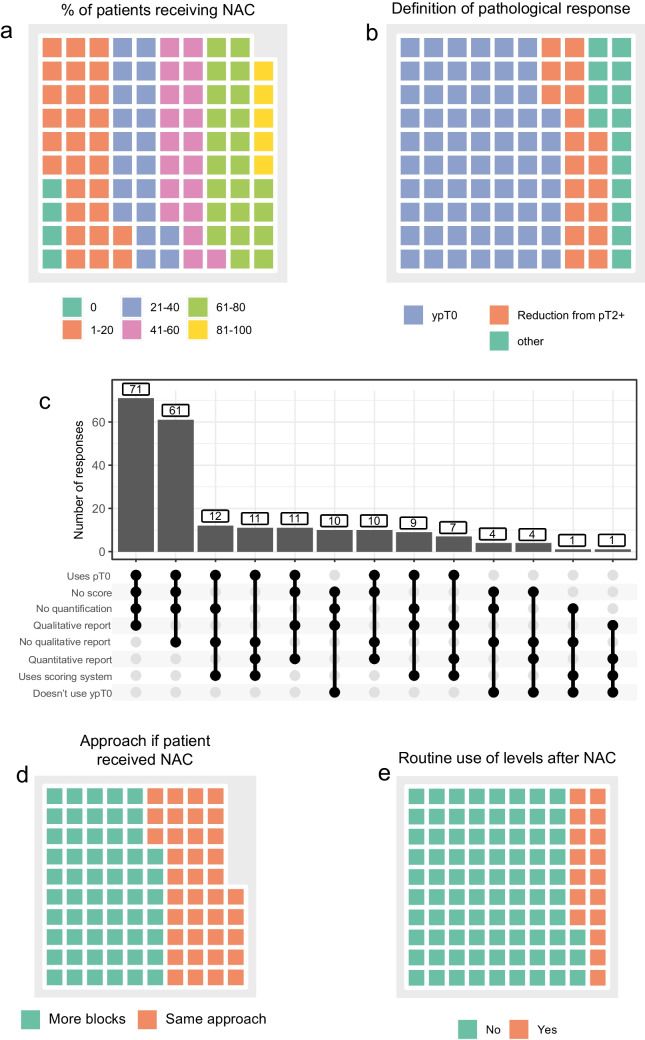
Approach to sampling and reporting cystectomy specimens after neoadjuvant chemotherapy. (**a**) Proportion of patients receiving neoadjuvant chemotherapy (NAC). Data from *n* = 144 respondents. Sixty-eight did not know what proportion of patients receive NAC in their centre. (**b**) Definitions of pathological response to NAC. Thirty respondents gave a descriptive answer. These responses are grouped together as ‘other’. (**c**) Upset plot of combinations of reporting practices when describing response to NAC. (**d**) Approach to block taking after a patient had received NAC. (**e**) Use of levels/step sections in cystectomy specimens following NAC

Next, we asked how pathologists approached reporting cystectomy specimens following NAC. Overall, 192/212 (90%) pathologists stated they would use the ypT nomenclature when reporting pathological complete response. In total, 19% of respondents reported using a response score such as that described by Fleischmann et al. [4, 5]. Interestingly, all of these pathologists were from institutions outside of the USA (41/122 from non-USA institutions vs. 0/49 from USA institutions, *p* < 0.001, *c*^2^ test). As participants could select more than one option when asked how they reported pathologic response, we next investigated if there were common combinations of reporting practice. Most respondents (71, 33.5%) used the ypT0 nomenclature together with a qualitative description of the response to neoadjuvant therapy. The next commonest reporting combination was to use only ypT0 nomenclature with no qualitative description, quantification or scoring system to characterise the response to neoadjuvant treatment (*n* = 61, 28.8%) (Fig. 3).

We also asked if NAC changed the pathologists’ approach to reporting cystectomies. Some 56% said they would take more blocks in this scenario whilst the remaining 44% would not change their approach. The reported routine use of levels or step sections was significantly higher in post-NAC cystectomies with 18% of respondents using routine levels after NAC compared to 10% in when NAC had not been given (*p* = 0.03, *c*^2^ test).

### Use of digital and molecular pathology in reporting cystectomies

As digital and molecular pathology have become established facets of modern pathology practice, we sought understand how these tools are used in the assessment of cystectomies. Some 86% of respondents report cystectomies using traditional glass slides. By contrast, only 8% and 6% of pathologists reported using digital slides for all or some of their cystectomy work respectively. Of the 29 pathologists of who use digital slides, 14 (48%) reported using digital tools such as digital measuring in their assessment of cystectomies.

**Fig. 4:**
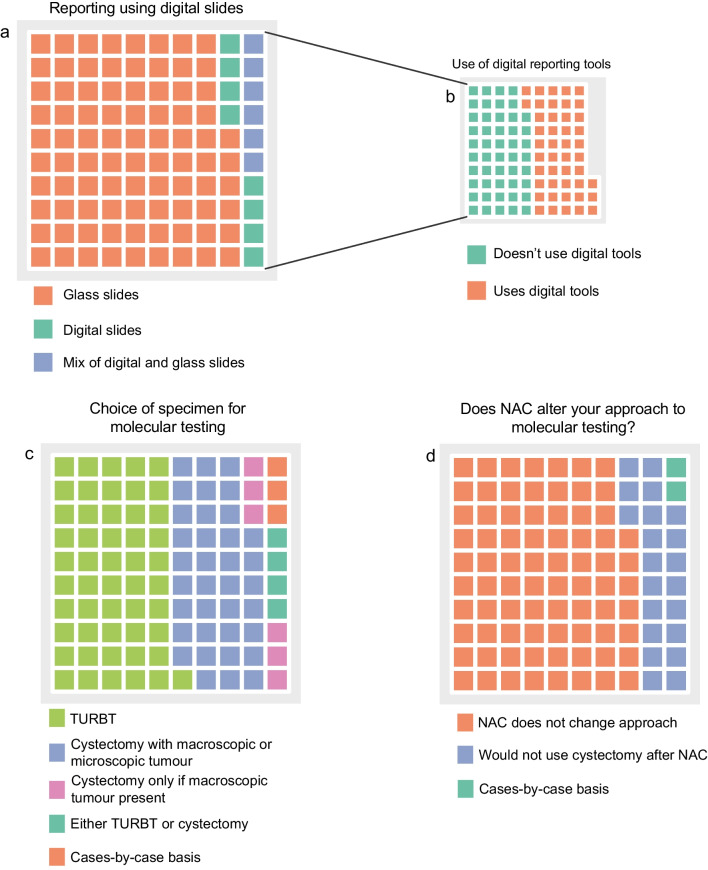
Use of digital and molecular pathology in radical cystectomy specimens. (a) Proportion of pathologists using glass slides, digitals slides and a mix for reporting. (b) Proportion of respondents who use digital slides or a mix of glass (*n* = 29) and digital who use digital tools for evaluating cystectomy specimens. (c, d) Use of TURBT and cystectomy specimens for molecular tests

Finally, we asked about molecular pathology reporting practice. There were 157 pathologists who answered the question and, of these, 52% preferred to perform molecular tests on the TURBT specimen. Thirty-five percent of respondents would do molecular tests on the cystectomy specimen if there was macroscopic or microscopic tumour present whereas 6% of pathologists would use the cystectomy but only if macroscopic tumour was visible (Fig. 4c). In the last question, we asked if NAC would change the molecular testing approach. The majority (140/181, 77%) of pathologists indicated that NAC would not change their approach to the choice of specimen for molecular testing. Some 20% of respondents would not use the cystectomy specimen for molecular testing if the patient had received NAC.

## Discussion

To our knowledge, this is the first international survey of cystectomy dissection and reporting practice. We found variation in practice across the entire specimen journey. Most pathologists open the bladder anteriorly and fix by submersion in formalin. This has the advantage of allowing fresh tissue sampling prior to fixation and is the method currently in use by the INVEST window of opportunity trial [8]. Formalin inflation via a catheter is also described in best practice guidelines [9, 10]; however, our data shows this not widely used. To our knowledge, there has not been a rigorous, head-to-head comparison of the two techniques. Proponents of formalin inflation claim that the urothelium undergoes rapid fixation, presumably with better resulting tissue preservation and microscopic morphology. However, this and the effect of each method on molecular testing have not been formally evaluated. In MIBC, any gains in diagnostic fidelity may be negligible when assessing grossly evident tumour. However, it is conceivable that microscopic foci of tumour following neoadjuvant therapy or in cystectomies for non-muscle invasive bladder cancer with prior intravesical BCG instillation may have variable appearances depending on fixation method.

There was broad consensus regarding block taking regardless of the presence of macroscopic tumour in the bladder. A small but significant minority of respondents described taking representative sections of scarred areas. A smaller proportion of pathologists indicated that they examined the entire bladder. A previous study showed that this approach did not change the detection of prognostically important parameters such as tumour stage [11]. Coupled with our findings, this suggests that representative sections and sampling of the entire scarred area where applicable is sufficient. We recognise that the wording of this part of the questionnaire does not allow us to distinguish between scenarios where the urothelium appears completely normal macroscopically or where a scarred area is present. Following fixation, it can be difficult to identify subtle macroscopic changes and so the distinction between normality and scar may not be reliable. This specific situation warrants further investigation. It may be that pathologists, when faced with a completely normal bladder macroscopically, are more likely to submit the entire specimen for microscopic evaluation. Whilst this was not an explicit option in the questionnaire, we did not receive feedback regarding this in the free text comments section. In addition, we not ask specifically about how pathologists used radiology to guide their sampling or the perceived benefits of radiological-pathological correlation. From the authors’ experience, pre-operative CT or MRI scans of the bladder can be useful in identifying the site of tumour after apparently complete TURBT.

We also assessed pathologist approach to resampling a specimen after initial microscopic assessment and found equipoise between the approaches of further sampling and no further sampling where no tumour was found in initial sections. This is a potential source of variability and should be evaluated prospectively. Furthermore, we demonstrated variability in the use of levels/step sections with more than half of pathologists using these on a case-by-case basis. It is important to note that extra sampling and levels still only provide a representative sample of the tissue examined and sampling error cannot be entirely excluded. However, there is likely to be a point at which sampling and levels approaches the limit of the useful information that could be achieved by complete examination. As complete examination (e.g. complete embedding and complete sectioning) is not feasible, exploration of the utility of sampling and levels is required. Our data show variation in the application of these tools, implying the optimum approach is not yet known.

Ours is the second survey to evaluate pathologists’ approach to post-NAC cystectomy specimens. Saunders et al. [12] surveyed 55 pathologists practicing in the USA via X (formerly Twitter). In agreement with our data, they also found that most pathologists submit the entire tumour bed area for assessment. Interestingly, respondents estimated tumour bed or scar only was a situation encountered in 71% of cases which is significantly higher than reported ypT0 rates following NAC [13]. Whilst presence of tumour bed or scar only does not directly translate to the absence of microscopic tumour, the discrepancy between macroscopic and microscopic impression implies a significant proportion of patients with microscopic-only residual tumour. Furthermore, ypT0 could represent complete resection by TURBT and limited contribution of NAC. These situations have not been evaluated in the literature to date and merit further study.

Our study adds to the evidence base of post-NAC cystectomy assessment. Interestingly, a third of respondents did not know what proportion of patients received NAC in their centre. We found that the complete absence of tumour in the bladder (ypT0) was the preferred definition of complete pathologic response (pCR); however, some pathologists also regarded downstaging as a pathologic response. We identified factors that could lead to variation in reporting pCR including variability in whether NAC changed pathologists’ approach to sampling and tissue submission. We also identified combinations of reporting practices when describing response to NAC. Nearly 20% of pathologists use the tumour response score described by Fleischmann et al. [4]; however, this was used exclusively by pathologists from outside of the USA. This system has been validated to predict overall survival following NAC; however, we could only find its inclusion in one recently published guideline from the Brazilian Societies of Pathology, Urology and Clinical Oncology [14]. Our data suggest that the uptake of this system has been low. This may reflect uncertainty over how this score might be used in clinical practice and if it would influence post-operative decision-making around adjuvant therapy.

pCR is widely used as a surrogate outcome measure in clinical trials of neoadjuvant therapies. Our survey results suggest that there is variation in how pCR is assessed and reported. This may in turn introduce variation into clinical trial results where pCR is an outcome. Recently, completed trials of neoadjuvant therapy in MIBC where pCR or pathological downstaging was an outcome include NEOBLADE [15], ABACUS [16] and NCT02812420 [17]. These trials define pCR as pT0 and used downstaging to < pT2 as a secondary outcome measure. However, protocols for fixation, processing and assessment of cystectomies were not documented in detail, and no central pathology review of pCR was mandated. Given the variability in practice we have highlighted in our survey responses, it is possible that these trial outcomes include variation from pathology practice that could mask or alter true therapeutic effect. Indeed, a recent position statement from the Society for Immunotherapy of Cancer and the International Bladder Cancer Group suggested that pT0/pCR may not be an appropriate sole primary endpoint in neoadjuvant trials [18].

Our study has some limitations. We used an electronic survey distributed via four major urological pathology societies. Whilst this resulted in the largest cohort of pathologists giving their opinion on cystectomy reporting to date, this approach may also have self-selection bias. This has previously been noted in patient surveys [19] and citizen science [20] projects. This bias should be taken into account when considering the generalisability of our findings. A further limitation was highlighted by the free comments section of the survey. Respondents suggested further areas for scrutiny including approaches to prostate sampling in cysto-prostatectomy, uterus and vaginal wall sampling in anterior exenteration, lymph node sampling and approaches to sampling cystectomies for non-muscle invasive bladder cancer. Our survey included 18 questions. Consideration of these additional areas may have made the survey more difficult to complete and affected the number of responses. We suggest that these areas are included in future work in this area.

In summary, we have demonstrated variability in cystectomy pathology reporting practices using an international survey of more than 200 pathologists. Clinical trials often use pathological measures of response to therapy as a surrogate endpoint but the variability of reporting practice could have an effect on the veracity of these measures and consequently the conclusions of clinical trials. The evidence base around cystectomy pathology reporting needs development and we have identified key research questions (Table 1). A Delphi survey would be a reasonable next step using the responses described in our questionnaire to inform statement design and expert discussion/ consensus. Delphi studies are underutilised in pathology but can provide useful information about current and best practice, and highlight areas for future research [21, 22]. This could be particularly useful for standardising the approach to histological response to NAC and this will become more important with greater use of neoadjuvant immunotherapy and small molecule inhibitors and ongoing trials investigating bladder sparing approaches [15, 16, 23]. Recently, there has been renewed interest in the evidence base underpinning macroscopic evaluation and specimen dissection and sampling [24, 25]. Development of evidence-based macroscopy and assessment of pCR have had clinical impact in colorectal [26–28] and breast cancer (29). We believe similar development of the evidence base for cystectomy sampling and reporting could be similarly useful.

**Table 1:** Research questions in cystectomy macroscopy, microscopy and reporting

Is there an optimal method of cystectomy fixation?
How should attached prostate be sampled?
How should lymph nodes be sampled?
Should there be different approaches for dissection of cystectomies performed for NMIBC and MIBC?
What is the role of large/ mega blocks in sampling the bladder and prostate in cystectomy specimens?
Is there benefit to using a tumour response score when evaluating post-NAC cystectomies?
Should a bladder with no microscopic tumour undergo further sampling?
How and when should levels/step sections be used in cystectomy assessment?
How can we standardise pathology processes and reporting for neoadjuvant clinical trials when pCR is an endpoint?
What is the role of digital pathology in reporting cystectomy specimens?

## Supplementary Information

Below is the link to the electronic supplementary material.

Supplementary file1 (DOCX 20 KB)

## Declarations

All participants gave informed consent to complete the questionnaire and the study received ethical approval from the University of Sheffield (UK) Ethics Committee on 24th July 2023 (approval number: 054611).
