# Supplementary material for: Variation in cystectomy pathology reporting practice—results from an international survey of 212 pathologists
Source: Virchows Arch. 2024 Sep 7;485(5):879–88. doi: 10.1007/s00428-024-03924-3 (PMC11564217; doi:10.1007/s00428-024-03924-3)
Supplement: Supplementary file 1 — Supplementary file1 (DOCX 20 KB) [file 428_2024_3924_MOESM1_ESM.docx]

**Supplementary information for Griffin et al., Variation in Cystectomy Pathology Reporting Practice – Results from an International Survey of 212 Pathologists**

**Cystectomy questionnaire**

A survey of how pathologists sample and assess radical cystectomy specimens.

**Participant information and consent**

You have been invited to complete this survey as a pathologist who reports radical cystectomy specimens as part of your routine practice. This survey is for pathologists who have completed their training and work in a substantive post (e.g. consultant or attending pathologists). Participation is voluntary. Please read the information below before deciding if you wish to take part. The questionnaire takes approximately 5 minutes to complete.

**What is the purpose of this research?**

Radical cystectomy can be a curative treatment option for muscle invasive bladder cancer and high risk non-muscle invasive bladder cancer. Guidelines for the macroscopic and microscopic assessment of these specimens are available however within the scientific literature numerous approaches have been described. 30-40% of patients receive neoadjuvant (pre-operative) chemotherapy and the response to this is often used as a surrogate endpoint in clinical trials. We aim to survey the different approaches used by pathologists in assessing cystectomy specimens, particularly with regards to assessing response to neoadjuvant (pre-operative) chemotherapy.

**Is my taking part in this study confidential?**

Yes. We are not collecting any identifiable/personal information and you do not need to provide an email address to take part.

**Do I have to take part in this study?**

No. Participation is voluntary.

**What are the benefits and risks of participating?**

There are no immediate benefits for participants however we hope that by collecting a wide range of responses we can understand how cystectomy reporting practice varies and whether there is any scope for standardisation. This approach has worked well in other cancers, for example colorectal cancer. There is minimal risk in participating. No personal data will be collected and all answers are anonymous. Data will be analysed and presented in aggregate form. No individual responses will be reproduced.

**What will you do with the survey data?**

The data will be stored securely at the University of Sheffield, UK. The data will be analysed to look for trends in cystectomy reporting practice. This may be published in academic journals of presented at conferences/meetings. No individual responses will be reproduced in any of these formats.

**Data Protection and General Data Protection Regulations**

Any data collected, stored or used for this research project will be done in accordance with the principles of GDPR regulations.

**Who has approved this survey?**

The University of Sheffield Research Ethics Committee has reviewed and approved this information and the survey (approval number: 054611)

**Who should I contact if I have questions or concerns?**

Please contact jonathan.griffin@sheffield.ac.uk if you have any questions.

This survey has been designed by:

Jon Griffin, Clinical Lecturer in Histopathology, University of Sheffield, UK

Eva Compérat, Professor of Pathology, Medical University of Vienna, Austria

Arndt Hartmann, Professor of Pathology, University Erlangen-Nürnberg

Thank you for taking the time to complete this survey.

1. I consent to take part in this study by completing the questionnaire

2. I have read and understood the information provided above

3. What country is your primary practice based in?

4. How long have you been reporting uropathology specimens?

5. How many cystectomies does your department report in a year?

1-10, 11-20, 21-30, 31-40, 41-50, 51-60, 61-70, more than 70

6. What percentage of your cystectomies have received neoadjuvant chemotherapy (please estimate to the nearest 10 % or select ‘don’t know’)?

Grossing/dissection, macroscopic assessment and sampling

7. How do you fix cystectomy specimens?

Inflate bladder with formalin via a catheter and submerge bladder in formalin

Make an incision in the bladder and submerge in formalin, leaving the bladder and attached organs largely intact

Cut bladder and attached organs into two halves and submerge in formalin

Other (please describe)

8. Do you perform fresh sampling of cystectomy specimens?

If yes, please describe how

9. How do you sample cystectomies with macroscopically visible tumour?

-Entire bladder is put into blocks

-Entire tumour is put into blocks plus background bladder sampling

-Entire tumour is put into blocks with no additional sampling of background bladder

-Representative samples of tumour are taken plus background bladder sampling

-Representative samples of tumour are taken with no additional sampling of background bladder

- Other (please describe)

10. How do you sample cystectomies with no macroscopically visible tumour?

-Entire bladder is put into blocks

-Entire scarred area is put into blocks plus background bladder sampling

-Entire scarred area is put into blocks with no additional sampling of background bladder

-Representative samples of scar are taken plus background bladder sampling

-Representative samples of scar are taken with no additional sampling of background bladder

- Other (please describe)

11. If a patient has received neoadjuvant chemotherapy do you change your approach to grossing/dissection of a cystectomy?

Yes – I take more blocks

Yes – I take fewer blocks

No – I sample all cystectomy specimens the same way

Other (please describe)

Microscopy

12. If you see no tumour microscopically would you block the rest of the bladder?

13. Do you routinely use levels/step sections to assess all cystectomies? If yes, on which blocks do you routinely perform levels.

14. Do you use levels/step sections specifically in cystectomies where neo-adjuvant chemotherapy has been given?

15. Do you use levels/step sections on a case-by-case basis? If yes, please give details about the situations where you would use levels.

16. How do you report response to neoadjuvant chemotherapy in cystectomy specimens (select all that apply)?

I report the stage with a ‘y’ prefix (e.g. ypT0)

I give a qualitative or descriptive estimate of residual tumour

I give a quantitative estimate of the tumour:stroma proportion

I give a quantitative estimate of the number of blocks containing tumour

I use a regression score (e.g. Fleischmann et al 2014. Am J Surg Path)

Other (please describe)

17. Do you report cystectomy specimens using digital slides or glass slides?

I report all cystectomies using glass slides

I report all cystectomies using digital slides

I use glass slides for some cases and digital slides for others

18. If you use digital slides for reporting cystectomies, do you use digital tools for quantifying residual tumour (e.g. ruler/ area measurement)?

I do not report cystectomies using digital slides

I report using digital slides and use digital tools for quantifying residual tumour

I report using digital slides and do not use digital tools for quantifying residual tumour

19. If doing molecular tests (PD-L1 immunohistochemistry, DNA mutation tests or gene expression profiling) which of the following statements most closely matches your practice?

I do molecular tests on the trans urethral resection of bladder tumour (TURBT) specimen

I do molecular tests on the cystectomy specimen only if there is macroscopic tumour

I do molecular tests on the cystectomy specimen if there is microscopic or macroscopic residual tumour

20. Does neoadjuvant chemotherapy change your practice in molecular testing?

Yes - if the patient has received neoadjuvant chemotherapy I would not perform molecular tests on residual tumour in the cystectomy specimen

No – I would do molecular tests on a cystectomy specimen with residual tumour whether or not neoadjuvant chemotherapy had been given

No – I wouldn’t do molecular testing on cystectomy specimens
